# Structures of two lyssavirus glycoproteins trapped in pre- and post-fusion states and the implications on the spatial-temporal conformational transition along with pH-decrease

**DOI:** 10.1371/journal.ppat.1012923

**Published:** 2025-02-19

**Authors:** Fanli Yang, Sheng Lin, Xin Yuan, Siqi Shu, Yueru Yu, Jing Yang, Fei Ye, Zimin Chen, Bin He, Jian Li, Qi Zhao, Haoyu Ye, Yu Cao, Guangwen Lu

**Affiliations:** 1 Department of Emergency Medicine, State Key Laboratory of Biotherapy, West China Hospital, Sichuan University, Chengdu, Sichuan, China; 2 School of Basic Medical Sciences, Chengdu University, Chengdu, Sichuan, China; 3 College of Food and Biological Engineering, Chengdu University, Chengdu, Sichuan, China; 4 Department of Biotherapy, Cancer Center and State Key Laboratory of Biotherapy, West China Hospital, Sichuan University, Chengdu, China; 5 Disaster Medicine Center, West China Hospital, Sichuan University, Chengdu, Sichuan, China; Icahn School of Medicine at Mount Sinai, UNITED STATES OF AMERICA

## Abstract

Lyssavirus glycoprotein plays a crucial role in mediating virus entry and serves as the major target for neutralizing antibodies. During membrane fusion, the lyssavirus glycoprotein undergoes a series of low-pH-induced conformational transitions. Here, we report the structures of Ikoma lyssavirus and Mokola lyssavirus glycoproteins, with which we believe that we have trapped the proteins in pre-fusion and post-fusion states respectively. By analyzing the available lyssaviral glycoprotein structures, we present a sequential conformation-transition model, in which two structural elements in the glycoprotein undergo fine-modulated secondary structural transitions, changing the glycoprotein from a bended hairpin conformation to an extended linear conformation. In addition, such conformational change is further facilitated, as observed in our surface plasmon resonance assay, by the pH-regulated interactions between the membrane-proximal region and the pleckstrin homology and the fusion domains. The structural features elucidated in this study will facilitate the design of vaccines and anti-viral drugs against lyssaviruses.

## Author summary

Lyssavirus, as represented by the prototypic rabies virus, is responsible for approximately 60,000 human fatalities annually. The glycoprotein of lyssavirus is a key factor mediating the low pH-induced membrane fusion of the viral envelope with the host cells. It is proposed that lyssavirus glycoprotein will experience a series of structural transitions in response to low environmental pH. Nevertheless, a detailed structural transition model remains to be atomically illustrated. In this study, we determined two crystal structures of lyssavirus glycoproteins at pH-8.3 and pH-4.0, which have trapped the glycoprotein in the pre-fusion and post-fusion states, respectively. By analysing the two newly resolved lyssavirus glycoprotein structures, together with two rabies virus glycoprotein structures reported previously, we identified two structural elements in the glycoprotein with fine-modulated secondary structural changes. Furthermore, we also proposed a detailed spatial-temporal structure-transition model for the glycoprotein along with pH-decrease, which could guide the vaccine and drug design against lyssavirus.

## Introduction

Lyssaviruses are a group of enveloped viruses taxonomically affiliated to the *Lyssavirus* genus of the *Rhabdoviridae* family [1,2]. Currently, 17 lyssaviruses have been identified in this genus [2], some of which can infect humans and other mammals, causing lethal acute encephalitis described as rabies or rabies-like diseases [1,3]. Lyssaviruses can be further subdivided into three phylogenetic groups (phylogroup-1, -2, and -3), among which the prototype virus, rabies virus (RABV), is classified as a phylogroup-1 member and is by far the most common causative agent for human rabies. This disease leads to nearly 100% mortality after the onset of clinical symptoms even with advanced supportive care. Human rabies is estimated to cause ~60,000 deaths worldwide each year and imposes a huge social burden [4,5]. In comparison to the classical RABV, the other 16 lyssaviruses are often referred to as rabies-related lyssavirus or non-RABV lyssavirus.

Glycoprotein (G), as the sole protein on the virion surface, is a key factor mediating lyssavirus invasion. It is also the major target for neutralizing antibodies and the crucial protein antigen for vaccine development. At the first stage of virus invasion, lyssavirus G is responsible for engaging receptors on host cells. Several potential cellular receptors/components have been identified for their ability to facilitate RABV infection [6–9]. After receptor engagement, lyssavirus enters the cell via an endocytic pathway, during which the G protein undergoes a series of pH-dependent conformational transitions to catalyze the fusion of the viral envelope with the endosomal membranes, thereby initiating the infection [10]. The lyssavirus G, which belongs to the rhabdovirus G family, has been defined as a class III viral membrane-fusion protein [11]. Along membrane fusion induced by low pH, studies in RABV have demonstrated that lyssavirus G could transit among different conformational states [11,12]. It has been proposed that these states exist as a pH-dependent thermodynamic equilibrium, such that it is shifted from the pre-fusion state towards the post-fusion state as pH descends [13–15]. This results in a reversible structural transition in lyssavirus G, differentiating it from the class I viral membrane-fusion glycoprotein (for example, influenza virus hemagglutinin and coronavirus spike), for which the low-pH-induced transition is primed by proteolytic cleavage and the transition is irreversible [11,12]. It is believed that such reversibility would allow the protein to recover its native pre-fusion conformation after its transport through the acidic compartments during secretion [16]. Members of the *Vesiculovirus* genus in the *Rhabdoviridae* family, as represented by the vesicular stomatitis virus (VSV), are also featured with reversible transitions in the glycoprotein [17–20]. These studies have implicated that lyssavirus G and vesiculovirus G are conformationally flexible on viral surface.

Facilitated by a series of high-resolution structures, detailed features underlying the pH-dependent structural transitions for vesiculovirus G have been well characterized [17–21]. The trimeric pre-fusion and post-fusion architectures are illustrated with VSV G (VSV-G) [17,18]. In addition, several structures proposed to have trapped the protein in different intermediate states are subsequently reported for another vesiculovirus, Chandipura virus (CHAV) [20,21]. These structures reveal that vesiculovirus G consists of three individual domains: a fusion domain (FD), a pleckstrin homology domain (PHD), and a central domain (CD). In response to low pH, large domain-rearrangements occur, leading the protein from a bended hairpin conformation into an extended linear conformation.

Structural characterization of lyssavirus G are attracting wide attention in recent years. The first atomic picture of the lyssavirus protein is depicted based on two ecto-domain structures of RABV G (RABV-G), which are proposed to have trapped the protein in an early-intermediate and a late-intermediate state, respectively [22]. Similar to its vesiculovirus homolog, RABV-G also consists of three domains: FD, PHD, and CD. These three domains arrange into an overall bended architecture in the early-intermediate structure and re-arrange into an extended architecture in the late-intermediate structure. Another study has reported the crystal structure for the ecto-domain of Mokola lyssavirus G (MOKV-G). The structure (named MOKV-G_1-436_*) also shows an extended conformation and seems to more resemble that observed in the post-fusion VSV-G [23]. It is interesting that the crystal of MOKV-G_1-436_* was obtained at pH 7.5, rather than at an acidic pH. Recently, the structures of trimeric pre-fusion RABV-G in complex with neutralizing antibodies have been determined by cryo-electron microscopy (Cryo-EM) [24,25].

In this study, we first prepared the G ecto-domain proteins of different lyssaviruses from all the three phylogenetic groups by the fusion-loop substitution strategy developed before [22]. Crystal structures of Ikoma lyssavirus G (IKOV-G) and MOKV-G were solved at pH-8.3 and pH-4.0, respectively. For IKOV-G crystallized at pH-8.3, the structure showed a bended conformation and a trimeric architecture resembling those observed in the Cryo-EM pre-fusion RABV-G structures, highlighting a similar trimeric pre-fusion architecture shared by lyssavirus G. For MOKV-G crystallized at pH-4.0, the structure exhibited a linear conformation similar to the previously reported MOKV-G_1-436_* structure solved at pH-7.5 [23] but was clearly much more extended than the latter, therefore featuring, in our opinion, as a more typical post-fusion configuration. Guided by the structures, we proposed a detailed spatial-temporal structure-transition model for lyssavirus G along with pH-decrease, which was further corroborated by the mutagenic analyses. Finally, we also observed an interesting low-pH-induced binding of the C-terminal region (CTR) of the G ectodomain with PHD-FD, which should also facilitate the pH-dependent structural transition of lyssavirus G. These structural and functional data will shed light on the mechanism of lyssavirus entry mediated by G and should be able to support vaccine and drug design.

## Results

### A universal strategy for preparing well-behaved lyssavirus G ectodomain protein

**Fig 1:**
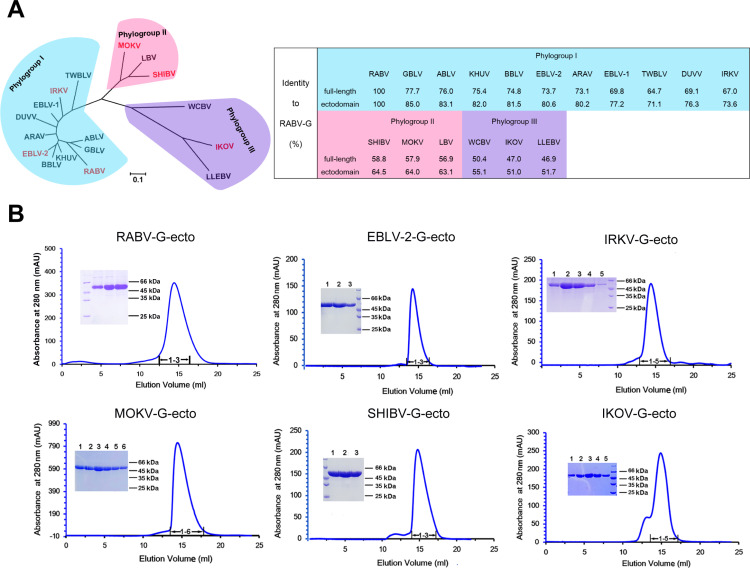
Preparation of lyssavirus glycoproteins. (A) The left panel shows a phylogenetic analysis of the 17 lyssavirus members within the *Lyssavirus* genus based on the glycoprotein amino acid sequences. Those members colored in red highlight the lyssaviruses for which their G-ecto proteins are prepared in this study. The right panel shows the identities of the indicated lyssavirus G to RABV-G based on the full-length or the ectodomain amino acid sequences. (B) Gel-filtration profiles of the purified lyssavirus G-ecto proteins on a Superdex 200 Increase 10/300 GL column. The inset figures show the SDS-PAGE analyses of the pooled samples.

The *Lyssavirus* genus encompasses 17 virus species which have been subdivided into three phylogroups (Fig 1A). We had previously developed a ‘fusion-loop substitution’ strategy for preparing G-ectodomain (G-ecto) protein of RABV with high yield and good behavior [22]. Here we applied the same strategy to prepare G-ecto proteins derived from non-RABV lyssaviruses. In comparison to RABV, non-RABV lyssaviruses share about 51.0-85.0% identities in the G-ecto sequences and about 46.9-77.7% identities in the full-length G sequences (Fig 1A). We selected representative members of all the three lyssavirus phylogenetic groups (including European bat 2 lyssavirus (EBLV-2), Irkut lyssavirus (IRKV), Mokola lyssavirus (MOKV), Shimoni bat lyssavirus (SHIBV), and Ikoma lyssavirus (IKOV)) and prepared their G-ecto proteins. Expectedly, the resultant protein preparations (RABV-G-ecto, EBLV-2-G-ecto, IRKV-G-ecto, MOKV-G-ecto, SHIBV-G-ecto and IKOV-G-ecto) all exhibited good solution behaviors when analyzed by gel filtration (Fig 1B). The result demonstrated that ‘fusion-loop substitution’ represented a universal strategy for preparing the G-ecto proteins of lyssaviruses.

### Pre-fusion structure of IKOV-G crystallized at pH-8.3

We successfully crystallized the IKOV-G-ecto protein at pH 8.3 and determined the structure at 2.9-Å resolution (S1 Table). In the crystallographic asymmetric unit, a total of six IKOV-G molecules, which are of essentially the same structure, are observed to assemble into two IKOV-G trimers. Because each protomer showed a similar topological structure to RABV-G [22], we therefore termed the individual domains and the secondary structural elements of IKOV-G accordingly as in the RABV-G structure reported in our previous work [22] (S1 Fig).

**Fig 2:**
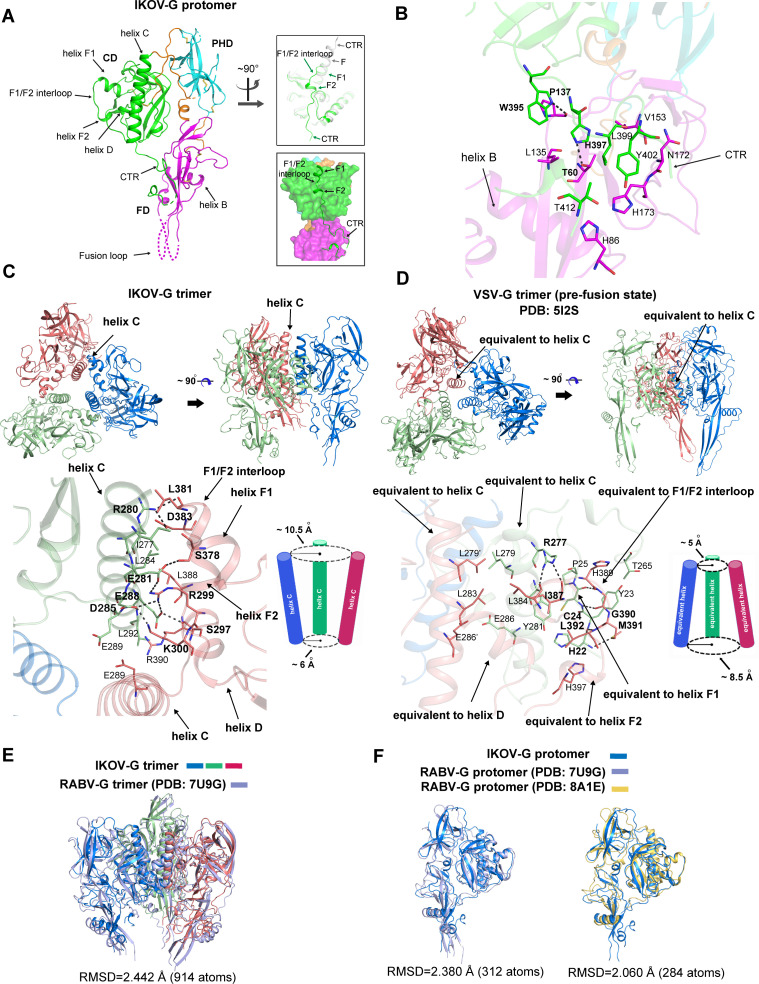
Structure of IKOV-G at pH-8.3. (A) The left panel shows an overview of the protomer structure of IKOV-G. Domains CD, PHD, and FD are colored in green, cyan, and magenta, respectively, and domain-linkers are colored in orange. Dashed lines indicate regions that are not traceable in the electron density map. The secondary structural elements are denoted as illustrated in S1 Fig. The top-right panel shows a superimposition of the IKOV-G structure with the RABV-G structure (derived at pH 8.0, colored in gray; PDB code:6LGX), highlighting conformational difference at CTR (C-terminal region of the molecule). The bottom-right panel highlights the steric position of CTR in our IKOV-G structure. (B) The atomic interactions between FD and CTR. Those amino acids referred to in the text are shown and labelled. Dashed lines indicate H-bonds and the related residues are marked with bold letters. (C and D) Comparison of the trimeric architecture observed in our IKOV-G structure (C) and the previously reported VSV-G structure (D). In each case, the top panels show an overview (a top view at the left and a side view at the right) of the architecture. The three protomers are colored in deep blue, pale green, and deep salmon, respectively. The central helices (helix C in IKOV-G and the equivalent helix in VSV-G) are marked. The bottom left panel illustrates the inter-protomer contacts stabilizing the trimeric architecture. Those residues referred to in the text are shown and labelled. The bottom right panel shows a schematic figure marking the distance between the central helices. Overall, the three helices are arranged as an inverse frustum-cone for IKOV-G and as a frustum-cone for VSV-G. The top and bottom radii are individually labelled. (E) Superimposition of our IKOV-G trimer structure (protomers colored in deep blue, pale green, and deep salmon, respectively) onto a previously reported pre-fusion trimer structure of RABV-G (PDB code: 7U9G, colored in sky blue). (F) Superimposition of our IKOV-G protomer structure (colored in deep blue) onto previously reported protomer structures of RABV-G [(PDB code: 7U9G, colored in sky blue) and (PDB code: 8ALE, colored in yellow)]. The calculated root mean square deviation (RMSD) values are labelled.

Although a certain portion of the FD domain remains untraceable because of poor electron densities, the majority of the IKOV-G residues are clearly visualized in the electron density map. Overall, the solved structure clearly shows a bended hairpin conformation. In comparison to the previously reported structure of RABV-G in the early-intermediate state, however, a remarkable difference was observed in the location of the helix F element and the following CTR. Rather than forming a single elongated helix F and extending out from FD in the intermediate conformation, the residues L380-H384 of IKOV-G adopt a loop conformation, resulting helix F into two short helices (F1 and F2) connected via an inter-helix loop (Figs 2A and S2A). The F1 helix stretches out and resembles its equivalent part of RABV-G (early-intermediate). The inter-loop and helix F2, however, turn back to facilitate the following CTR loop being trapped in a surface groove on FD (Fig 2A and 2B). The segment composed of W395-T412 in CTR interacts with FD, both through hydrogen bonds (H-bonds) and hydrophobic force (Fig 2B). Specifically, the nitrogen atom in imidazole group of residue H397 makes an H-bond with the side-chain -OH group of T60, representing a key factor of the CTR-FD interactions. Additionally, the side-chain of W395 also makes an H-bond with the main-chain of P137. Furthermore, a cluster of residues, comprising H86, L135, V153, N172, H173, W395, L399, Y402 and T412, are observed to converge together to provide hydrophobic interactions. It is notable that homologous interactions between FD and C-terminal region of G ectodomain are also reported in VSV-G [26,27].

While the G-ecto proteins prepared above are monomers in solution, IKOV-G forms biologically relevant trimers during crystallization. Within the trimeric architecture, three protomers are converged together via the CD domain (Fig 2C), with three C-helices almost juxtaposed to each other to form an inverse frustum-cone with top and bottom radii of about 10.5 Å and 6 Å, respectively (Fig 2D). It seems to be a rather loosened assembling mode. Among the juxtaposed C-helices, only few residues (eg. E289) locating at the cone bottom are observed to make inter-helix contacts via their side-chains. Instead, most inter-protomeric interactions arise from the contacts between helix C in one protomer and helix D and the F1-interloop-F2 element in the neighboring protomer (Fig 2C). The trimeric-interface is mainly supported by a cluster of salt-bridge interactions and H-bonds. Specifically, residues E281, D285, E288 and R280 in helix C, are observed to form a salt-bridge and H-bond network with residues R299, S378, K300, S297, D383 and L381 locating at the helix D, helix F1 and helix F2 of the neighboring protomer (Fig 2C). In addition, the inter-protomer binding is further corroborated by a small number of apolar and van der Waals (vdw) contacts, mainly involving residues I277, L284, E289 and L292 in one protomer with L381, L388 and R390 in the neighboring protomer (Fig 2C).

The pre-fusion structure of VSV-G has also been determined as a trimeric architecture assembled around the three central helices (Fig 2D) [18]. Forming an obvious contrast to those observed in the IKOV-G trimer, however, the three helices are packed in a more compact manner, arranging into a frustum-cone with a top-radius of about 5 Å and a bottom-radius of about 8.5 Å. Accordingly, the three protomers in the VSV-G trimer are more intimately stacked against each other, which are stabilized by hydrophobic and vdw contacts among three central helixes (involving residues L279, L283, E286 and L384) and the intimate interactions between mainly the central helix in one protomer and the structural elements equivalent to IKOV-G’s helix D and F1-interloop-F2 in the neighboring protomer (involving H22-P25, T265, R277, Y281 in one protomer and amino acids L384, I387, H389-L392, H397 in the other protomer) (Fig 2D).

In comparison to the previously reported structures of pre-fusion RABV-G determined by Cryo-EM [24,25], IKOV-G exhibits a similar trimeric architecture (Fig 2E). The protomer structures of IKOV-G and RABV-G could also be well superimposed (Fig 2F), despite that the two glycoproteins share the lowest sequence identity (~51% for their ectodomains) in the *Lyssavirus* genus (Fig 1A). Taken together, these results indicate that we have solved the structure of IKOV-G in the pre-fusion state.

### Post-fusion structure of MOKV-G crystallized at pH-4.0

**Fig 3:**
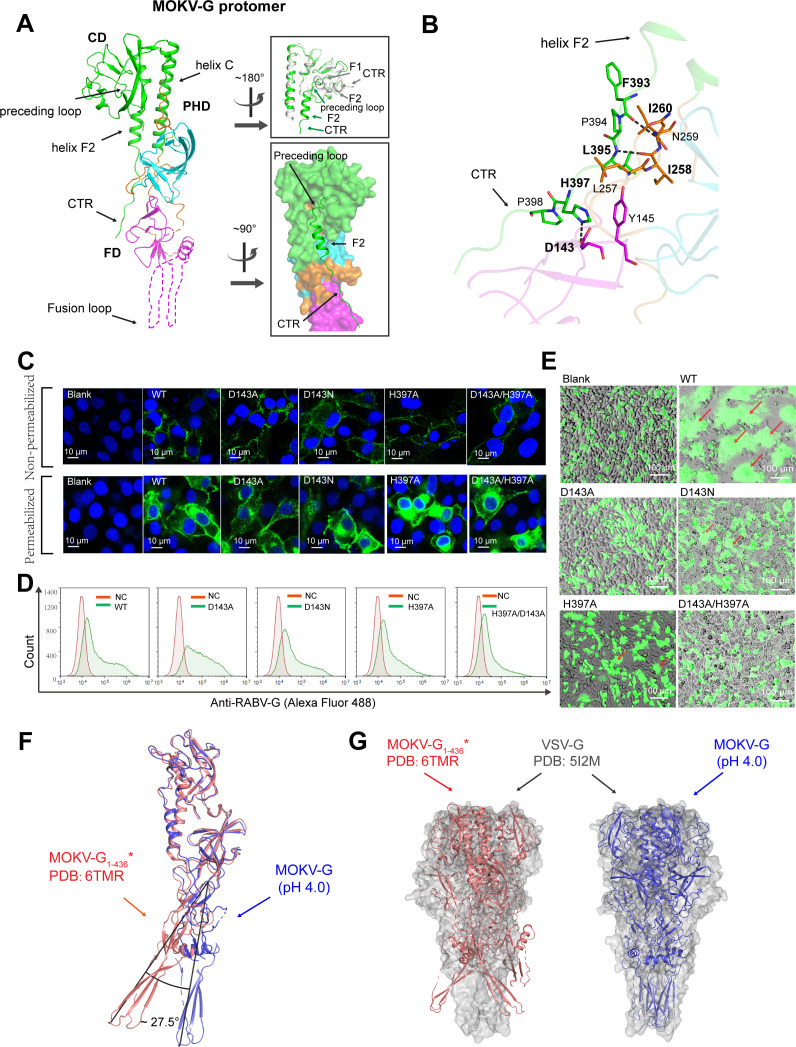
Structure of MOKV-G at pH-4.0. (A) The left panel shows an overview of the structure of MOKV-G. Domains CD, PHD, and FD are colored in green, cyan, and magenta, respectively, and domain-linkers are colored in orange. Dashed lines indicate regions that are not traceable in the electron density map. The secondary structural elements are denoted as illustrated in S1 Fig. The top-right panel shows a superimposition of the MOKV-G structure with the RABV-G structure (derived at pH 6.5, colored in gray; PDB code: 6LGW), highlighting conformational difference at CTR. The bottom-right panel highlights the steric position of CTR in our MOKV-G structure. (B) The atomic interactions between FD and CTR. Those amino acids referred to in the text are shown and labelled. Dashed lines indicate H-bonds or salt-bridges, and the related residues are highlighted with bold letters. (C) Representative cell images showing the proper cell-surface location of the indicated glycoproteins. Transfected BHK-21 cells were pre-treated with or without permeabilization, and the glycoprotein was detected by immunostaining. Scale bar: 10 μm. (D) Quantitative analysis of the glycoprotein surface expression in BHK-21 by flow cytometry. (E) Representative cell images illustrating the variant capacity of the indicated glycoproteins to induce syncytium-formation. For clarity, cells are co-transfected to allow for simultaneous expression of EGFP and RABV-G. Single GFP is used as a negative control. The red arrows mark the formed syncytia. Scale bar: 100 μm. (F) Superposition of our MOKV-G structure (deep blue) onto a previously reported MOKV-G_1-436_* structure (PDB code: 6TMR, deep salmon) based on CD. The domain-orientation differences observed in FD are marked. The invisible portion of the FD domain in our MOKV-G structure is rebuilt with the equivalent portion of the FD domain from MOKV-G_1-436_* and is colored in light blue. (G) The left panel shows superposition of the MOKV-G_1-436_* structure (deep salmon) onto VSV-G structure (grey) based on CD. The right panel shows superposition of our MOKV-G structure (deep blue) onto VSV-G structure (grey) based on CD. The trimeric post-fusion structure of VSV-G (PDB code: 5I2M) is shown in surface representation, and the MOKV-G structures are shown in cartoon representation.

We also crystallized MOKV-G-ecto at pH 4.0 and determined the structure at 3.2 Å resolution (S1 Table). Three monomers were present in the asymmetric unit but were not arranged in a trimer. They interact via crystal contacts that are unlikely of biological relevance. The CD and PHD, as well as a majority of the FD amino acids, are successfully defined in the electron-density map (Fig 3A). The overall domain-arrangement mode in the structure is similar to that observed in the post-fusion structure of VSV-G, as indicated by the extended linear conformation and the proximity of CTR to FD (Fig 3A and 3B) [17,20]. Compared to the previously-reported late-intermediate structure of RABV-G (PDB code: 6LGW) [22], MOKV-G exhibits distinctive features in the helix-F element (Figs 3A and S2B). Helix F1 is unfolded as a preceding loop, orienting back towards the PHD and FD domains. This allows the F2 helix to be arranged in parallel to helix C (Figs 3A and S2B). Consequently, the CTR loop is shown to extend over domains PHD and FD, enabling multiple amino acid interactions between CTR and PHD-FD along the interface. The CTR residue H397 forms a salt-bridge with D143 in FD. Additionally, CTR residues F393 and L395 are observed to form main-chain H-bonds with I260 and I258, respectively. Finally, hydrophobic interactions among residues Y145, L257-I260, P394, L395 and P398 also contribute to stabilize the post-fusion architecture (Fig 3B).

To verify the functional importance of the CTR/PHD-FD interactions observed in our structure, we focused on the salt-bridge residues D143 and H397 which exhibited a high degree of conservation among *Lyssavirus* glycoproteins (S1 Fig). Of note, an equivalent salt-bridge interaction has also been observed in VSV-G (formed by D137 and H407), and H407 has been identified as a key player in structural transition [27]. Consequently, we generated four mutants of RABV-G (D143A, D143N, H397A, and D143A/H397A) and analyzed their capacities to induce syncytia formation in parallel with the wild-type (WT) glycoprotein. In BHK-21 cells, WT, D143A and D143N mutant glycoproteins are all efficiently expressed and properly transported to the cell surface (Fig 3C). Using flow cytometry, we further showed that WT, D143A and D143N mutant glycoproteins featured with similar levels of cell surface location (Fig 3D). For mutations of H397A and D143A/H397A, however, the H397A mutant and to a lesser extent the D143A/H397A mutant were less transported than WT G to the surface. Furthermore, the H397A mutant were also observed to be accumulated more in the endoplasmic reticulum (Fig 3C and 3D). These observations coincided well with the corresponding mutant in VSV G (the H407A mutant) which was also shown to be less transported to cell surface [27]. Regarding the syncytium-formation, the results showed that single mutation of D143A and double mutation of D143A/H397A could completely abolish the G-mediated cell-cell fusion (Fig 3E). For the D143N (which would abolish the salt bridge with H397 but likely still allow for H-bonds) and H397A mutants, the formation of cell-syncytia was also clearly inhibited, with a dramatic decrease in both the fusion-cell numbers and the syncytium size (Fig 3E). These results demonstrated that disruption of the D143/H397 interactions could severely impact, even completely block, G-mediated membrane fusion, which in turn highlighted the indispensable role of the CTR/PHD-FD interactions in lyssavirus G activity.

We also compared our MOKV-G structure with a previously reported structure of MOKV-G_1-436_* (PDB code: 6TMR), which also showed an elongated architecture [23]. When the two structures were superimposed based on the CD domain, we observed a large domain-orientation difference (by ~27.5°) for FD. Resultantly, our structure displayed apparently a much more extended linear-conformation than the MOKV-G_1-436_* structure (Fig 3F). We further aligned the two MOKV-G structures to the trimeric post-fusion structure of VSV-G (PDB code: 5I2M). As expected, our MOKV-G structure, with a much more extended conformation, could be well modeled into the post-fusion VSV-G trimer architecture. In contrast, the FDs of MOKV-G_1-436_* were observed to extend away from the central axis of the VSV-G trimer (Fig 3G). These findings suggest that the complete post-fusion structure of lyssavirus G is similarly characterized by a straight-linear conformation as observed in the vesiculovirus homologs, which in turn indicates that we have solved the structure of MOKV-G in the post-fusion state (Fig 3G). Accordingly, the crystal used in our study was obtained at the acidic pH of 4.0, whereas the MOKV-G_1-436_* structure was reported to be derived at pH 7.5 [23].

### Stepwise structure-transitions for lyssavirus G induced by low pH

**Fig 4:**
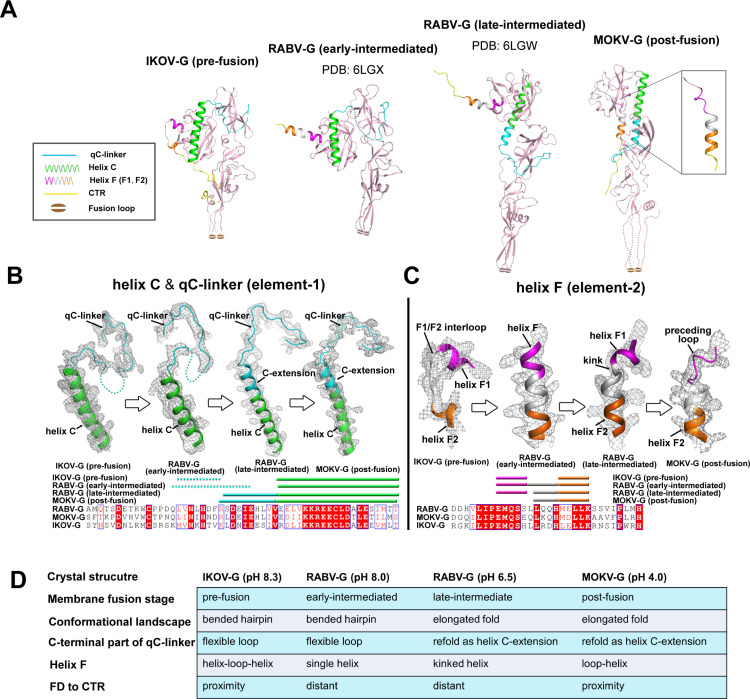
Stepwise transitions during low-pH induced structural transitions mediated by lyssavirus G. (A) The two lyssavirus G structures solved in this study, along with previously reported RABV-G structures, are aligned in a pH-descending manner. Two structural elements [element-1 (composed of qC-linker and its following helix C) and element-2 (composed of helices F1 and F2, and their interloop)] are highlighted. (B) Structural transitions in element-1 with a multiple sequence alignment at the bottom using horizontal cylinders for helices. The electron densities are contoured at 1.0 σ using the 2 | Fo | - | Fc | map. Dashed lines indicate regions that are not traceable due to flexibility. (C) Structural transitions in element-2 with a multiple sequence alignment at the bottom using horizontal cylinders for helices. The electron densities are contoured at 1.0 σ using the 2 | Fo | - | Fc | map. (D) The structural features for lyssavirus glycoprotein at each stage are summarized and listed.

To gain a systematic view on the pH-dependent structural transition process of lyssavirus G, we further aligned the above-solved two lyssavirus G structures (IKOV-G and MOKV-G) together with two intermediated RABV-G structures [22] along an axis in a pH-descending manner (Fig 4A), which revealed two structural elements that experience remarkable structure refolding (Fig 4). The element-1 is qC-linker (the loop linking strand q to helix C). In the basic-pH structures of IKOV-G and RABV-G (PDB code: 6LGX), this interloop is of high flexibility such that a number of the loop residues are density-untraceable (Fig 4A and 4B). In the acidic-pH structures of MOKV-G and RABV-G (PDB code: 6LGW), however, part of the loop is refolded into a helical structure, assembling into helix C-extension (an extension to helix C). In addition, such a transition is concomitantly accompanied by the large domain rearrangement, which leads the G-molecule from a bended hairpin conformation into an extended linear conformation (Fig 4A, 4B and 4D). It is notable that similar loop-to-helix transition induced by low pH has also been observed in the equivalent loop-linker in the VSV-G [28].

The element-2 (helix F) is composed of the short helices F1 and F2 and their inter-helix loop (Fig 4A and 4C). In the IKOV-G structure, the F1/F2 interloop remains a loop conformation, whose flexibility enables the FD domain to contact the CTR loop of the molecule. In the RABV-G structure (PDB code: 6LGX), this inter-helix loop refolds into a helical structure, uniting the F1 and F2 helices into a single long helix F. Resultantly, the FD domain and the CTR, as observed in the RABV-G structure at early-intermediated state, are separated from each other. As shown in the RABV-G structure (PDB code: 6LGW) at late-intermediated state, a kink appeared in the F helix. The relative rigidity of helices, however, makes domain FD remain distant from the CTR loop. Finally, at post-fusion state as observed in the MOKV-G structure, the F1 helix dis-assembled into a loop, allowing FD to be in the proximity to the CTR again (Fig 4A, 4C and 4D). The structure comparison therefore revealed a 2-step loop-helix transition-mode in qC-linker and a 4-step loop-helix-kink-loop transition-mode in helix F, respectively.

### Evidence of pH-regulated interactions between PHD-FD and CTR

**Fig 5:**
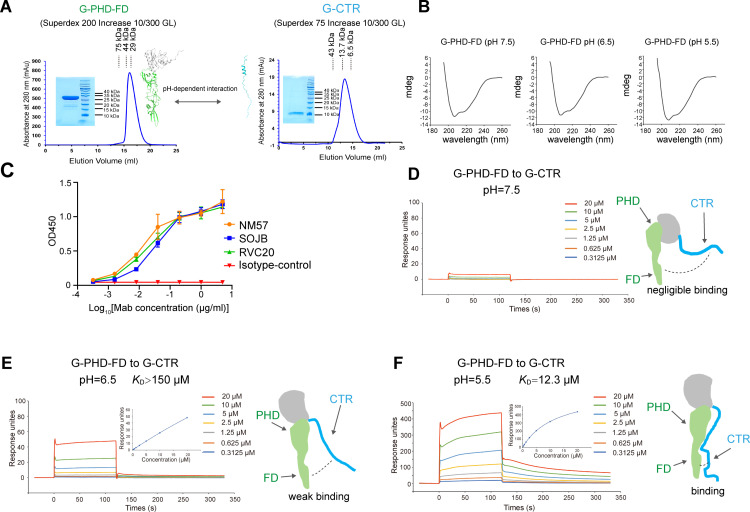
Characterization of the interactions between CTR and PHD-FD of RABV-G in solution at the indicated pHs. (A) Solution behavior of G-PHD-FD on a Superdex 200 Increase 10/300 GL column and G-CTR on a Superdex 75 Increase 10/300 GL column. The inset figure shows the SDS-PAGE analyses of the pooled samples. (B) CD spectra of the G-PHD-FD at pH 7.5 (left), pH 6.5 (middle) and pH 5.5 (right). (C) Binding profiles between G-PHD-FD and the indicated antibodies by ELISA. (D-F) Characterization of the interactions between RABV-G CTR and PHD-FD at pH 7.5 (D), pH 6.5 (E) and pH 5.5 (F) using surface plasmon resonance (SPR).

In addition to the stepwise structural transitions for the qC-linker and the F helix, the structures also showed that PHD-FD might associate with or dissociate from CTR during the structural transition process. We therefore set out to investigate if PHD-FD might interact with CTR in a pH-dependent mode. The PHD-FD domain protein (designated as G-PHD-FD) and the CTR loop protein (designated as G-CTR) derived from RABV-G were individually prepared to homogeneity (Fig 5A). The circular dichroism (CD) profile of G-PHD-FD featured with clear negative peaks at ~208 nm and ~217 nm, respectively. Analyses of the spectra using BeStSel (https://bestsel.elte.hu/index.php) revealed mixed secondary structural elements for the protein composed of low content of α-helices and high content of β-strands, coinciding well with the expected structure. We also performed the CD experiments at different pHs, which revealed quite similar and consistent spectrum profiles along pH-decrease (Fig 5B). Furthermore, we selected three PHD-specific antibodies (NM57, SOJB and RVC20) [29–31] and performed the antibody-binding test with G-PHD-FD using ELISA. As expected, our protein can be effectively recognized by all the three antibodies (Fig 5C). These results indicated the proper protein folding of G-PHD-FD. Consequently, the real-time interactions between G-PHD-FD and G-CTR were then characterized at different pHs via surface plasmon resonance (SPR). At pH 7.5, only negligible binding was observed (Fig 5D). When pH decreased to 6.5, we observed clear interactions between G-PHD-FD and G-CTR (Fig 5E). The affinity, however, was estimated to be >150 μM, representing a weak binding at pH 6.5. At pH 5.5, much more intimate interactions between G-PHD-FD and G-CTR were recorded and the affinity was determined to be ~12.3 μM (Fig 5F). Taken together, these results reveal an interesting pH-regulated binding of PHD-FD to CTR such that the binding affinity increases significantly along with pH-decrease.

It is worth noting that, the affinity between PHD-FD and CTR may be weak because the two entities are expressed separately. However, in the context of the complete G, when the two fragments are linked within the same protomer, the interaction may be much more stable. It had been proved that RABV-G in their post-fusion structure are observable between pH 6.7 and 6.4 on the surface of viruses [14–16].

## Discussion

It is believed that lyssavirus G would assemble into trimeric spikes on viral surface in either pre-fusion or post-fusion state. Intriguingly, we have obtained a crystal structure of IKOV-G displaying a trimeric pre-fusion architecture. In comparison to that observed in the pre-fusion trimer-structure of VSV-G, the trimerization-arrangement for IKOV-G is rather loosened and should therefore be of less stability (Fig 2C and 2D). Echoing these observations, previous studies have shown that RABV-G trimer is not as stable as VSV-G and can be easily disrupted using detergents [32,33]. It is also notable that two recent studies have reported the Cryo-EM structure of RABV-G in complex with pre-fusion-trimer stabilized antibodies [24,25]. The work has revealed the structural features of trimerized RABV-G in the pre-fusion state. The IKOV-G structure determined in our study presents a very similar architecture to that of the pre-fusion RABV-G trimer (Fig 2E and 2F). Noted that IKOV-G and RABV-G possess the lowest sequence identity in the *Lyssavirus* genus, we would therefore suggest that other lyssavirus G, which all has a higher sequence identity to RABV-G, shares a resembled pre-fusion trimer structure [24,25].

We also pay attention to the potential inter-molecule interactions between neighboring trimers in our IKOV-G structure. Though numerous inter-molecule contacts could be observed, they are not shown to directly affect the protomer assembly within the trimer. In addition, comparison our IKOV-G structure with previously reported pre-fusion-trimer structures of RABV-G solved by Cryo-EM (which would not subject to crystal packing effects) reveals quite similar trimer formation patterns. Thus, we believe that the trimeric architecture observed in our structure, which is quite different from that observed in the VSV-G structure, is unlikely due to crystal packing but a reflection of variant protomer-assembly mode between lyssaviruses and vesiculoviruses.

During lyssavirus entry, membrane fusion is accompanied with the low-pH induced structural transitions in G [1,10]. It is interesting that the IKOV-G and MOKV-G structures solved in this study, as well as the two RABV-G structures reported previously [22], are derived at different pHs. When the four G-structures were aligned in a pH-descending manner, we observed a series of sequential element-refolding events, which coincide well with the fusion process (Figs 4 and S3).

Step-1. With an overall bended hairpin conformation, the pre-fusion lyssavirus G is further featured with a relative short helix C and a flexible loop following the helix, as well as a helix-loop-helix structure for the helix F element. Such structural features would enable helix F1, the F1/F2 inter loop and helix F2 to establish extensive contacts with helix C of the neighboring protomers, stabilizing the pre-fusion trimeric architecture (Figs 2C and S3). Nevertheless, lyssavirus G assembles into pre-fusion trimers in a rather loosened manner. In addition, the interactions between FD and CTR are overall negligible at basic- or neutral-pH (Fig 5). Resultantly, the pre-fusion trimer of lyssavirus G is easily disrupted and would equilibrate with G in the early-intermediate state at step-2. In support of this, there have been studies showing that glycoprotein on the rhabdovirus surface is actually polymorphic in the neutral environment such that the G trimers and monomers could both be observed [19,21].

Step-2. Lyssavirus G undergoes de-trimerization due to the instability of its pre-fusion architecture and/or the decrease of the environmental pH (S3 Fig). The absence of constraints from neighboring protomers enables the F1-interloop-F2 element to re-unit into a single helix (helix F), sufficing lyssavirus G for domain re-arrangements in the next stage. While G remains a bended hairpin conformation, the early-intermediate structure likely has snapshot the protein in a state when the fusion loops are partially exposed (Figs 4 and S3).

Step-3. Further decrease of pH will cause large domain-rearrangements in G, leading to an extended linear conformation for the protein. Concomitantly, part of qC-linker will refold into a helical structure as an extension of helix C (or helix C-extension), preparing for the subsequent interactions with helix F in the next stage (Figs 4 and S3). Furthermore, a kink occurs in the F helix, featuring the start of the helix dis-assembly for the F1 region. The FD domain and the protein CTR are positioned almost on the opposite sides at this stage, thereby enabling the full exposure of the fusion loops for insertion into the target membranes (Figs 4 and S3).

Step-4. Proceeding into post-fusion stage, helix F1 will be completely unfolded into a loop, enabling helix F2 to be aligned in parallel with and thereby to interact with helix C-extension (Figs 4 and S3). Furthermore, the low environment-pH will also significantly increase the interactions between PHD-FD and CTR (Fig 5). Subsequently, CTR is locked to PHD-FD, dragging the viral envelope and the cell membrane into close proximity where fusion occurs. This represents the final stage of the structural transitions (S3 Fig).

Taken together, the structures have featured a multi-step spatial-temporal transition model for lyssavirus G in response to low-pH during membrane fusion. Nevertheless, it is worth noting that the proposed model is based on the static structures reported thus far. It cannot be ruled out that some missing intermediates are revealed in the future. In addition, a post-fusion-trimer structure for RABV-G or any other lyssavirus G remain unresolved. Though structure of MOKV-G obtained in our study is conformationally similar to the protomer structures of CHAV and VSV G in the trimeric post-fusion state [17,20], the MOKV-G molecule in our structure do not assemble into trimeric architectures. In such a context, the current structural transition model presumably still has limitations and might be optimized based on new lyssavirus G structures, which should be explored in the future.

Finally, a total of 17 viral species have been identified within the *Lyssavirus* genus [1,2]. Apart from RABV, the other 16 lyssaviruses can also cause fatal encephalitis resembling rabies. Currently, there is no vaccines available against non-RABV lyssaviruses. The inactivated vaccines against RABV are well-developed but with high cost, preventing widespread use in low-income countries. It is thus necessary to develop safe, effective and affordable subunit vaccines against RABV and non-RABV lyssaviruses. In this study, we successfully obtained well-behaved proteins of lyssaviruses G-ecto from all the three phylogenetic groups via the fusion-loop substitution strategy developed previously [22]. We believe the strategy for preparing lyssavirus G, which could act as vaccination immunogens, will pave the way for development of subunit vaccines against lyssaviruses.

## Materials and methods

### Cell lines

Sf9 (*Spodoptera frugiperda*; Invitrogen 11496015) and Hi5 (*Trichoplusia ni;* Invitrogen B85502) cells were individually maintained in the SIM SF medium and the SIM HF medium (Sino Biological) using a non-humidified shaker at 27°C. BHK-21 cells (Baby Hamster Kidney Cells; ATCC CCL-10) were maintained at 37°C in a humidified environment with 5% CO_2_ in DMEM medium supplemented with 10% FBS.

### Cloning, expression and purification

The RABV-G-ecto, EBLV-2-G-ecto, IRKV-G-ecto, MOKV-G-ecto, SHIBV-G-ecto and IKOV-G-ecto proteins used for crystallizations were prepared using the Bac-to-Bac baculovirus expression system (Invitrogen) as previously described [22]. The coding sequences (codon optimized for insect cells) of the full-length glycoproteins for the indicated RABV CVS-11 strain (GenBank: ADJ29911.1), EBLV-2 (GenBank: ABO65251.1), IRKV (GenBank: AAR03480.1), MOKV (GenBank: AAB26292.1), SHIBV (GenBank: ADD84510.1) and IKOV (GenBank: AFQ62097.1) were synthesized by the GENEWIZ Corporation and GENERAL BIOL Corporation. Constructs of Lyssavirus G (RABV-G-ecto, EBLV-2-G-ecto, IRKV-G-ecto, MOKV-G-ecto, SHIBV-G-ecto and IKOV-G-ecto) covered ecto-domain residues, along with replacing residues 73-79 and 117-125 with the G-G-S-G-G linker, respectively (S1 Fig). The coding sequence for RABV G-PHD-FD (used for SPR experiments) covered residues 28-270 (the amino acids in 73-79 and 117-125 were also replaced with the G-G-S-G-G linker). The above-mentioned constructs were individually sub-cloned into the pFastBac1 vector. For each construct, a previously described gp67 signal peptide sequence [34] was added to the protein N-terminus for protein secretion, and a 6×His tag was added to the C-terminus to facilitate further purification processes. Transfection and virus amplification were conducted with Sf9 cells, and the recombinant proteins were produced in Hi5 cells. Protein purification was performed using the protein purification system SDL-030-F2 (Sepure Instruments Inc.). The cell culture supernatants were collected 96 hours after infection and passed through a 5-ml HisTrap excel column (Cytiva) for primary purification. The recovered proteins were further purified on a Source 15Q column (Cytiva) and then on a Superdex 200 Increase 10/300 GL column (Cytiva). Finally, the proteins were exchanged into the buffer consisting of 20 mM Tris-HCl (pH 8.0) and 150 mM NaCl for further use.

The RABV G-CTR protein used for SPR experiments was expressed using *E.coli* expression system. The DNA fragment of G-CTR (residues 373-439) was first engineered to contain a coding sequence for an N-terminal maltose binding protein (MBP) tag, a PreScission Protease (PSP) cleavage site, and a C-terminal 6×His tag. The fused sequence of MBP-G-CTR was then subcloned into the pET-21a (Novagen) vector via the *Nde* I and *Xho* I restriction sites. The resultant recombinant plasmid was then transformed into *E. coli* BL21 (DE3), and the expression was conducted with the addition of 500 μM isopropyl-β-D-thiogalactoside (IPTG) followed by induction at 16°C for ~12 h. For protein purification, the *E. coli* cells were harvested, lysed by sonication in a re-suspension buffer composed of 20 mM Tris-HCl (pH 8.0) and 150 mM NaCl, and clarified via centrifugation at 18,000 × g for 20 min. The protein supernatant was passed through the Ni-NTA Beads (Cytiva), washed with the re-suspension buffer containing 20 mM imidazole, and then eluted with re-suspension buffer supplemented with 300 mM imidazole. The eluted target protein was then further purified by gel-filtration chromatography in PBS buffer using a Hiload 16/60 Superdex 200 pg (Cytiva) column. The MBP-fusion protein was digested by PreScission Protease in PBS buffer supplemented with 1 mM DTT and the cleaved proteins were passed through the Dextrin Beads (Smart-Lifesciences Biotechnology) to remove MBP tag. The RABV G-CTR protein in flow-through was further purified by gel-filtration chromatography in PBS buffer using a Superdex 75 Increase 10/300 GL column (Cytiva).

The recombinant full-length antibodies were prepared as previously described [22]. Briefly, the constant-region sequences containing a human immunoglobulin IgG1 heavy chain (GenBank: CAA75030.1) and human immunoglobulin light chain (GenBank: CAA75031.1) were cloned into the pCAGGS vector to yield the backbone plasmids pCAGGS-HC and pCAGGS-LC, respectively. The VH and VL sequences of NM57 (GenBank: AY172957.1 and AY172960.1), SOJB (GenBank: AY172958.1 and AY172962.1) and RVC20 (PDB: 6TOU) were then first engineered to include an IL-2 signal peptide coding sequence and then sub-cloned into pCAGGS-HC and pCAGGS-LC by homologous recombination, generating the final plasmids encoding the heavy chain and light chain of the lgG1 antibodies. For antibody expression, the heavy chain plasmid and light chain plasmid were co-transfected into HEK293T cells. The supernatants containing the recombinant antibodies were harvested respectively and subjected to HiTrap rProtein A FF column (Cytiva) for purification.

### Crystallization

Crystals were obtained by vapour-diffusion sitting-drop method with 1 μl protein mixing with 1 μl reservoir solution and then equilibrating against 90 μl reservoir solution at 18°C. The initial crystallization screenings were carried out using the commercial crystallization kits (Hampton Research and Molecular Dimensions). Crystals of IKOV-G-ecto were obtained from an optimized condition containing 0.2 M Ammonium phosphate dibasic (pH 8.3) and 20% w/v polyethylene glycol 3350 with a protein concentration of 7.5 mg/ml. Crystals of MOKV-G-ecto were obtained under a condition consisting of 0.1 M sodium acetate trihydrate (pH 4.0) and 10% w/v polyethylene glycol 4000 with a protein concentration of 7.5 mg/ml.

### Data collection and structure determination

For data collection, all crystals were flash-cooled in liquid nitrogen after a brief soaking in reservoir solution supplemented with 20% (v/v) glycerol. Diffraction data were collected at Shanghai Synchrotron Radiation Facility (SSRF) BL18U1 and BL19U1 [35] beamlines. All data were processed with HKL2000 [36] for indexing, integration and scaling. Structures were all solved by molecular replacement using PHASER [37] with the individual domains of the solved RABV-G structure [22] as the search models. Finally, the atomic models were completed with COOT [38] and further refined with PHENIX [39]. The final data processing and structure refinement statistics are summarized in S1 Table. All structural figures were generated using PyMOL (http://www.pymol.org).

### Cell-cell fusion assay for syncytia formation

Sequences encoding the wild-type (WT) full-length RABV-G (CVS-11 strain), and the RABV-G mutant (D143A, D143N, H397A and D143A/H397A) were subcloned into pCAGGS vector to yield the expression plasmids (WT and mutant pCAGGS_RABV-G). For expression, BHK-21 cells were pre-seeded on 12-well plates for overnight culturing. When 80% confluency was obtained, the cells were co-transfected with pEGFP-N1 (0.3 μg/well) and the WT or the mutant pCAGGS_RABV-G plasmids (0.3 μg/well). Cells transfected with pEGFP-N1 alone were used as the negative control. After 24 hours, cells were treated with the fusion buffer (20 mM MES in DMEM, pH 5.5) for 10 minutes at 37°C. The Cells were then washed with DMEM containing 20 mM HEPES (pH 7.0) once, followed by incubation in DMEM culture medium at 37°C for 1 hour. Finally, the cells were visualized for syncytia formation by fluorescence microscope (Olympus).

### Immunofluorescent assay verifying surface-location of WT and mutant RABV-G

BHK-21 cells were seeded on glass coverslips in 24-well plates. The cells (on glass coverslip) were transfected with the WT or the mutant pCAGGS_RABV-G plasmids. The blank pCAGGS plasmid was transfected serving as the negative control. Cells were fixed for 30 min with 4% PFA (paraformaldehyde) 24 hours post-transfection and then blocked for 1 hour at room temperature with PBS containing 5% skimmed milk. For permeabilization, cells were treated in 0.1% Triton X-100 in PBS for 20 min. Cells (with or without permeabilization) were incubated with the full-length antibody SOJB [22] at room temperature for 2 hours, followed by washing and incubation with Alexa Fluor 488 conjugated goat anti-human IgG (BBI) for 1.5 hours. Following washing, cells were incubated with DAPI solution to stain nucleus. Images were visualized by LSM 880 (Zeiss).

### Flow cytometry assay

To learn the surface location of the indicated glycoproteins in BHK-21, the cells were pre-seeded on 12-well plates for overnight culturing. When 80% confluency was obtained, the cells were transfected the WT or the mutant pCAGGS_RABV-G plasmids. After 24 hours, cells were digested by trypsin (Thermo Fisher) and the digestion stopped with DMEM (containing 10% FBS). Cells were centrifuged at 200 g, then resuspend in PBS (containing 1% BSA). Cells were incubated with full-length antibody SOJB at 4°C for 1 hour, followed by washing and incubation with Alexa Fluor 488 conjugated goat anti-human IgG (BBI) at 4°C for 1 hour. Finally, cells were washed with PBS and were monitored by flow cytometry (ACEA NovoCyte).

### Indirect ELISA

To learn the binding between G-PHD-FD and monoclonal antibodies, 96-well microtiter plates (Corning) were coated with purified G-PHD-FD at 100 ng/well in 0.05 M carbonate-bicarbonate coating buffer (pH 9.6) overnight at 4°C. The wells were then blocked for 1 hour at room temperature with PBS containing 5% skimmed milk. After blocking, 5-fold serially-diluted full-length antibodies (NM57, SOJB, RVC20 and CV3-25 [40] as an isotype-control) were added to the wells and incubated for 1 hour, followed by the addition of goat anti-human IgG-HRP (Merck Millipore) and incubation for another 1 hour. In each step, the plates were fully washed with PBST. The chromogenic reaction was conducted at room temperature for 3 minutes and was stopped with 2 M HCl. The emission OD450 was monitored using a microplate reader (Thermo). Three independent experiments were conducted and the data was graphed using GraphPad Prism 8.

### CD spectroscopy

Freshly prepared G-PHD-FD protein was divided into three portions and then respectively changed into the buffers with different pHs (pH 7.5, 6.5 or 5.5) containing 5 mM HEPES and 50 mM NaCl. The protein concentration was adjusted to 0.2 mg/ml. Circular dichroism (CD) spectra were collected on Chirascan (Applied photophysics) at 25°C using a 0.1-cm-path-length cuvette. Data was obtained by taking data points every 1 nm with a bandwidth of 1 nm. The secondary structure analysis was performed by BeStSel [41] using wavelength spectra ranging from 195 nm to 260 nm.

### Surface plasmon resonance (SPR) assay

SPR experiments were performed with the Biacore 8K system (Cytiva). G-CTR protein was immobilized to the CM5 sensor chip (Cytiva) with about 300 response units. Before the experiments, purified RABV G-PHD-FD protein was divided into three portions and respectively changed into the buffers containing with different pHs (pH 7.5, 6.5 or 5.5) through ultrafiltration. Serial dilutions of the G-PHD-FD protein were flowed over G-CTR protein for affinity determination in the running buffer containing 20 mM HEPES-NaOH (pH 7.5, pH 6.5 or pH 5.5), 150 mM NaCl and 0.05% Tween-20 at a rate of 30 μl/min. The obtained kinetic data were analyzed with the Biacore Insight Evaluation Software for dissociation constant calculations using the steady-state affinity model for the fast-on/fast-off data.

## Supporting information

S1 Fig Structure-based multiple sequence alignment of the glycoproteins from representative lyssaviruses. The signal peptide, the individual domains and the domain-linkers defined based on the structures, and the transmembrane and cytoplasmic domains are marked above the sequence. The secondary structural elements, denoted in alphabetical order along the protein sequence using the lowercase letters for β-strands (indicated with horizontal arrows) and uppercase letters for α-helices (indicated with spinal lines), are labelled above the sequence. The domains to which each strand and helix belong are marked in parentheses. The two fusion loops that are individually substituted with the G-G-S-G-G linker during protein preparation are highlighted in black squares. The D143 and H397 residues are marked by black arrows. The green square brackets mark the boundary residues selected for expression of G-ecto proteins derived from RABV, EBLV-2, IRKV, SHIBV, MOKV and IKOV in insect cells. Abbreviations: RABV (rabies virus), KHUV (Khujand lyssavirus), BBLV (Bokeloh bat lyssavirus), ARAV (Aravan lyssavirus), EBLV-1 (European bat 1 lyssavirus), EBLV-2 (European bat 2 lyssavirus), TWBLV (Taiwan bat lyssavirus), IRKV (Irkut lyssavirus), DUVV (Duvenhage lyssavirus), ABLV (Australian bat lyssavirus), GBLV (Gannoruwa bat lyssavirus), LBV (Lagos bat lyssavirus), SHIBV (Shimoni bat lyssavirus), MOKV (Mokola lyssavirus), WCBV (West Caucasian bat lyssavirus), IKOV (Ikoma lyssavirus), LLEBV (Lleida bat lyssavirus).
(TIF)

S2 Fig Comparison of the lyssavirus G structures reported in this study with previously reported RABV-G structures in intermediate states. (A) Structural superimposition of the IKOV-G protomer (green) onto the RABV-G protomer (orange) proposed to be trapped in the early-intermediate state. (B) Structural superimposition of the MOKV-G protomer (deep blue) onto the RABV-G protomer (pink) proposed to be trapped in the late-intermediate state.
(TIF)

S3 Fig A proposed spatial-temporal transition model for low-pH induced membrane fusion mediated by lyssavirus G. The three protomers are colored green, cyan and magenta, respectively. Those structural elements described in the text, such as helix C, qC-linker and helix F, are colored the same as in Fig 4.
(TIF)

S1 Table Crystallographic data collection and refinement statistics. (DOCX)
