# Supplementary figures and images for: Structures of two lyssavirus glycoproteins trapped in pre- and post-fusion states and the implications on the spatial-temporal conformational transition along with pH-decrease
Source: PLoS Pathog. 2025 Feb 19;21(2):e1012923. doi: 10.1371/journal.ppat.1012923 (PMC11864512; doi:10.1371/journal.ppat.1012923)

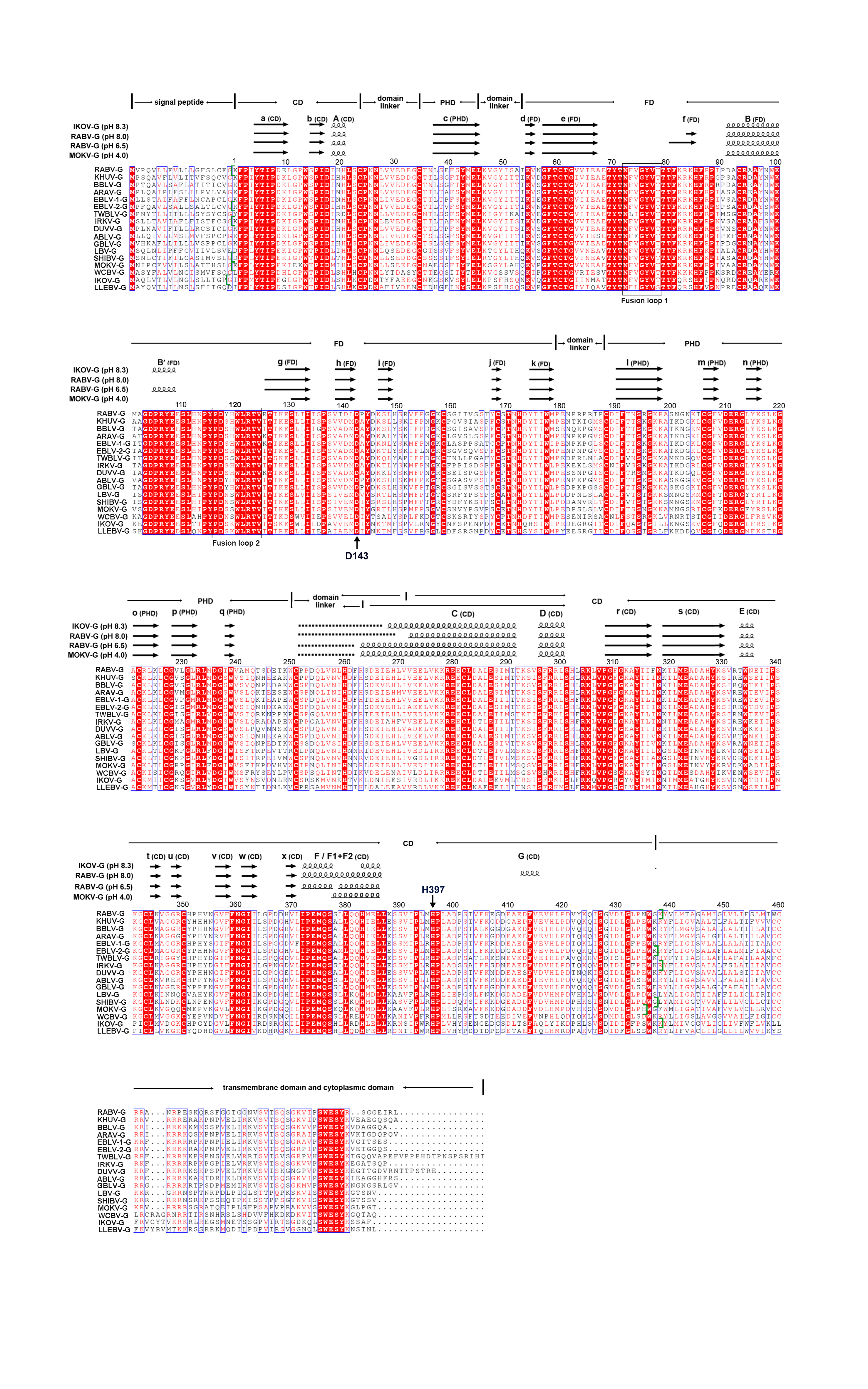

Supplement: S1 Fig — The signal peptide, the individual domains and the domain-linkers defined based on the structures, and the transmembrane and cytoplasmic domains are marked above the sequence. The secondary structural elements, denoted in alphabetical order along the protein sequence using the lowercase letters for β-strands (indicated with horizontal arrows) and uppercase letters for α-helices (indicated with spinal lines), are labelled above the sequence. The domains to which each strand and helix belong are marked in parentheses. The two fusion loops that are individually substituted with the G-G-S-G-G linker during protein preparation are highlighted in black squares. The D143 and H397 residues are marked by black arrows. The green square brackets mark the boundary residues selected for expression of G-ecto proteins derived from RABV, EBLV-2, IRKV, SHIBV, MOKV and IKOV in insect cells. Abbreviations: RABV (rabies virus), KHUV (Khujand lyssavirus), BBLV (Bokeloh bat lyssavirus), ARAV (Aravan lyssavirus), EBLV-1 (European bat 1 lyssavirus), EBLV-2 (European bat 2 lyssavirus), TWBLV (Taiwan bat lyssavirus), IRKV (Irkut lyssavirus), DUVV (Duvenhage lyssavirus), ABLV (Australian bat lyssavirus), GBLV (Gannoruwa bat lyssavirus), LBV (Lagos bat lyssavirus), SHIBV (Shimoni bat lyssavirus), MOKV (Mokola lyssavirus), WCBV (West Caucasian bat lyssavirus), IKOV (Ikoma lyssavirus), LLEBV (Lleida bat lyssavirus). (TIF) [file ppat.1012923.s001.tif]

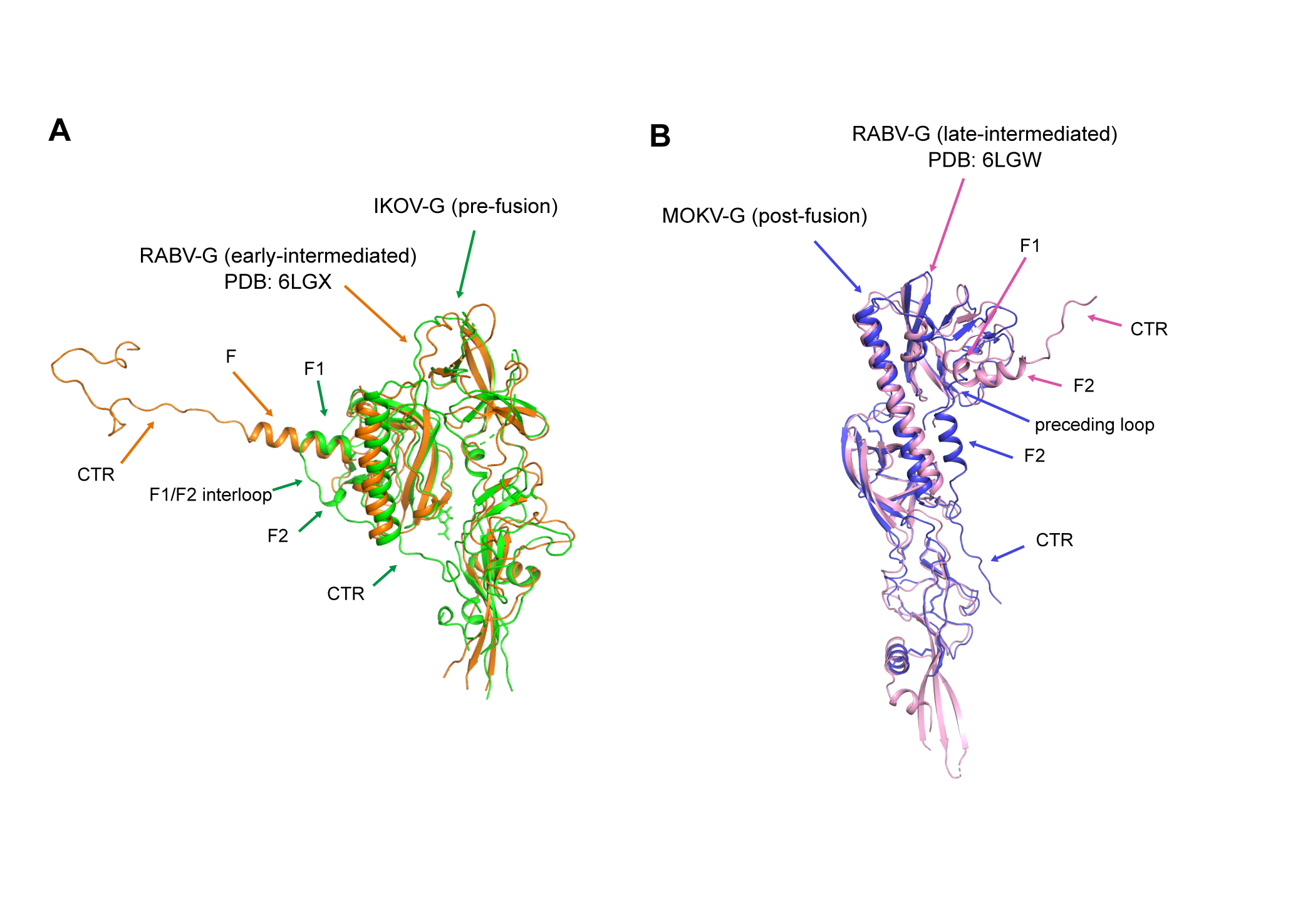

Supplement: S2 Fig — (A) Structural superimposition of the IKOV-G protomer (green) onto the RABV-G protomer (orange) proposed to be trapped in the early-intermediate state. (B) Structural superimposition of the MOKV-G protomer (deep blue) onto the RABV-G protomer (pink) proposed to be trapped in the late-intermediate state. (TIF) [file ppat.1012923.s002.tif]

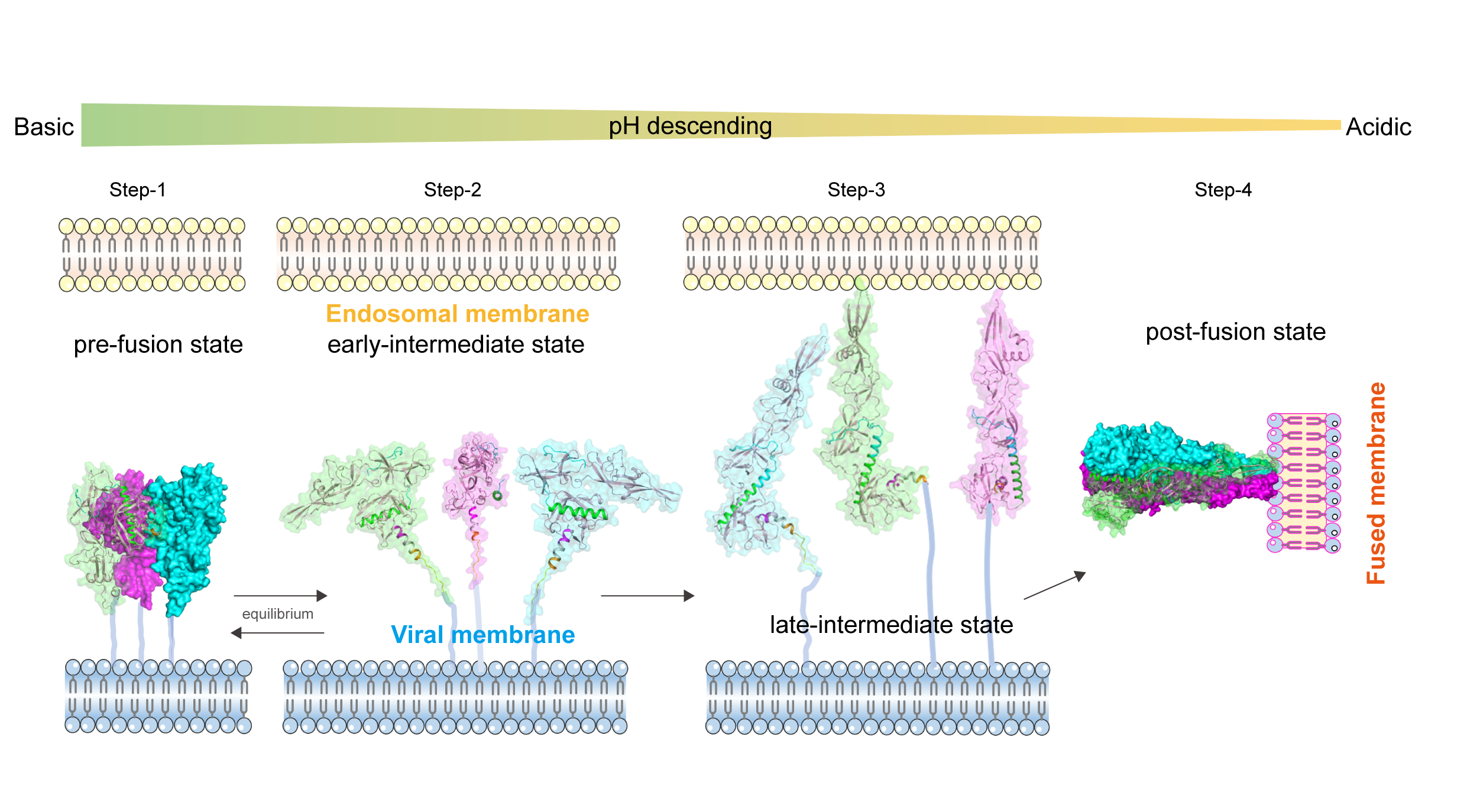

Supplement: S3 Fig — The three protomers are colored green, cyan and magenta, respectively. Those structural elements described in the text, such as helix C, qC-linker and helix F, are colored the same as in Fig 4. (TIF) [file ppat.1012923.s003.tif]
