# Supplementary material for: Structures of two lyssavirus glycoproteins trapped in pre- and post-fusion states and the implications on the spatial-temporal conformational transition along with pH-decrease
Source: PLoS Pathog. 2025 Feb 19;21(2):e1012923. doi: 10.1371/journal.ppat.1012923 (PMC11864512; doi:10.1371/journal.ppat.1012923)
Supplement: S1 Table — (DOCX) [file ppat.1012923.s004.docx]

**S1 Table.** **Crystallographic** **data collection and refinement statistics**

|  | IKOV-G | MOKV-G |
| --- | --- | --- |
| **Data collection** |  |  |
| Space group | P21 | P2 |
| Cell dimensions |  |  |
| *a*, *b*, *c* (Å) | 122.92, 90.75, 151.31 | 104.24, 87.52, 111.19 |
| α, β, γ (°) | 90.0, 90.0, 90.0 | 90.0, 111.9, 90.0 |
| Resolution (Å) | 50-2.90 (3.00-2.90) | 50-3.20 (3.31-3.20) |
| *R*_merge_ | 0.168 (0.961) | 0.115 (1.651) |
| *I* / σ*I* | 9.15 (1.35) | 14.58 (0.92) |
| Completeness (%) | 99.6 (99.5) | 95.2 (88.0) |
| Redundancy | 5.1 (5.2) | 7.2 (7.1) |
|  |  |  |
| **Refinement** |  |  |
| Resolution (Å) | 46.64-2.88 | 48.37-3.20 |
| No. reflections | 74334 | 29436 |
| *R*_work_ / *R*_free_ | 0.2251/0.2812 | 0.2465/0.2877 |
| No. atoms |  |  |
| Protein | 17726 | 7217 |
| Ligand/ion | 182 | 0 |
| Water | 0 | 0 |
| *B*-factors |  |  |
| Protein | 71.39 | 138.08 |
| Ligand/ion | 82.52 | 0 |
| Water | 0 | 0 |
| R.m.s. deviations |  |  |
| Bond lengths (Å) | 0.011 | 0.004 |
| Bond angles (°) | 1.46 | 0.750 |
| Ramachandran plot (%)  Favored region  Allowed region  Outlier region | 93.5  5.8  0.7 | 94.8  5.2  0.00 |
| **PDB code** | 8ZHZ | 8ZHW |

*In each case, a single crystal was used to collect the data. Values in parentheses are for the highest-resolution shell. Values in parentheses are for the highest-resolution shell.
